# Efficacy of Nutritional Strategies on the Improvement of the Performance and Health of the Athlete: A Systematic Review

**DOI:** 10.3390/ijerph19074240

**Published:** 2022-04-01

**Authors:** J. Javier Perez-Montilla, Maria Cuevas-Cervera, Ana Gonzalez-Muñoz, Maria Carmen Garcia-Rios, Santiago Navarro-Ledesma

**Affiliations:** 1 Department of Physiotherapy, Faculty of Health Sciences, Campus of Melilla, University of Granada, Querol Street, 5, 52004 Melilla, Spain; perezmontilla@correo.ugr.es (J.J.P.-M.); maaricuevass@correo.ugr.es (M.C.-C.); 2 Clínica Ana González, Avenida Hernan Nuñez de Toledo 6, 29018 Malaga, Spain; 3 Department of Physiotherapy, Faculty of Health Sciences, University of Granada, 18071 Granada, Spain; mcgrios@ugr.es

**Keywords:** intermittent fasting, sport, caloric restriction, fasting, ketogenic diet, time-restricted feeding

## Abstract

Evidence shows that the use of food strategies can impact health, but a clear consensus about how the effects of different food strategies impact improvement in the athlete’s performance and health remain unclear. This study evaluated how food strategies, specifically intermittent fasting and a ketogenic diet affect health and performance in healthy athletes. Study selection for this review was based on clinical trial studies analyzing changes in performance and health in athletes. The Pubmed, Web of Science, PEDro, Dialnet, Scopus, CINAHL, ProQuest, Medline and Cochrane databases were searched. The Physiotherapy Evidence Database (PEDro) scale, PEDro Internal Validity Scale (IVS) and Standard Quality Assessment Criteria for Evaluating Primary Research Papers from a variety of fields (QUALSYT) checklists were used to evaluate the risk of bias of the included studies. Articles were selected based on criteria concerning the effectiveness of nutritional strategies on athletes’ performance; articles should be randomized clinical trials (RCTs) or uncontrolled clinical trials; they should be human studies and they should have been published less than 7 years ago. A total of 15 articles were evaluated, 8 randomised clinical trials and 7 non-randomized clinical studies, with 411 participants who satisfied our inclusion criteria and were included in this review. The results of the study showed intermittent fasting and time-restricted feeding as strategies that produce health benefits. On the other hand, the ketogenic diet did not reach an appropriate consensus. The articles presented a medium level of methodological quality in the PEDro scale, low quality in IVS scale and high quality in QUALSYT scale. Despite the lack of studies analyzing changes in the performance and health of athletes after the use of different nutritional strategies, intermittent fasting and time-restricted feeding should be considered since they seem to be effective, and further studies are necessary.

## 1. Introduction

Lifestyle factors such as nutrition, poor sleep, smoking, stress, unhealthy diet, and obesity/overweight have been shown to have an impact on health and chronic conditions [1]. Among such factors, nutrition is considered to be one of the most important, with current research being steered towards the study of the effect of diet on pain and systemic inflammation biomarkers [2].

Nutrition influences health depending on the type of diet and the amount of daily intake. The quality as well as the quantity of food that is ingested has an impact on the metabolic and molecular health of the organism [3,4]. To achieve an improvement and have an influence on health, different nutritional strategies such as caloric restriction (CR), intermittent fasting (IF) and ketogenic diet (KD) are used. An increasing body of evidence from basic research points to the existence of beneficial effects of intermittent and periodic fasting in chronic conditions [5,6].

In the sports field, all these aforementioned nutritional strategies are an important subject within sports and nutrition sciences, since their ability to increase performance benefits in athletes remains controversial. It is known that nutritional requirements for athletes are high, even in the most optimized subjects; total body glycogen stores (500 g, yielding approximately 2000 kcal of energy if completely oxidized) would be insufficient to supply the necessary energy for endurance events such as an Iron Man triathlon, which is estimated to require almost 9000 kcal of energy to complete. In contrast, even very lean athletes store massive amounts of energy (63,000 kcal or more) in adipose tissues [6]. To achieve enough muscle and liver glycogen stores, a high CHO diet has been traditionally promoted for athletes in order to maximize their performance, as well as their ability to keep their efficacy [7]. However, low-carbohydrate diets offer a ‘metabolic advantage’ by increasing total energy expenditure. When comparing different methods, substantial increases in average daily energy expenditure have been found during low-carbohydrate diets when compared with high-carbohydrate diets, as measured by the doubly labelled water method, although such results appear to be inconsistent [8], and are opposite to the recommendations from international organizations such as the International Olympic Committee, the Academy of Nutrition and Dietetics, the American College of Sports Medicine, and Dieticians of Canada, which indicate that athletes with high training loads should consume a high-carbohydrate diet [9].

It is also of great importance to point out differences in the effects of nutritional strategies, such as IT, CR and KT, in aerobic or anaerobic exercises. The mild systemic acidity caused by KD might predispose the muscle to more rapidly develop more severe acidosis during high-intensity anaerobic exercise, thereby inhibiting muscle contractile function and impairing performance. In this scenario, it would be expected that high-intensity, short-duration exercise performance would be lower when athletes use KD as compared to high-carbohydrate diets. On the other hand, KD mayincrease performance in endurance sports [10].

In this regard, the body of research is increasing, but more studies are needed to increase the knowledge to answer questions such as the specific nutritional requirements for athletes when using different strategies, when it is best to use each strategy, and whether those strategies are safe for athletes or not. In all cases, it is currently recommended that athletes should undertake an audit of their event and their personal experiences to balance the risk of impaired performance in their activity [10].

The caloric restriction has been shown to greatly reduce the segregation of hormonal and metabolic factors involved in biological deterioration due to aging [11]. Another factor reduced through caloric intake is low-grade inflammation, which is characterized by appearing during increases in the concentration of inflammatory markers without the need of other symptoms. This type of inflammation is associated with poor physical activity, being overweight, tobacco use and poor eating habits. Other benefits of decreased caloric intake have been shown, such as an increase in life expectancy, delayed onset of aging-related diseases, avoiding or preventing age-related brain deficits, increasing visual cortex plasticity, and improving cognitive function [12,13,14,15,16].One of the diets based on caloric restriction is intermittent fasting, which is a dietary regimen consisting of periods in which caloric intake is reduced and others in which a normal diet is followed [13]. The objective of this regimen is to induce a reduction in net energy intake that makes it fall below energy expenditure, thus creating a negative energy balance state that induces weight loss, among other results [17]. This practice has emerged as an effective therapeutic strategy to improve multiple cardiometabolic endpoints, ranging from reducing weight or body fat, improving insulin sensitivity, reducing glucose and insulin levels, lowering blood pressure, improving lipid profiles, and reducing biomarkers of inflammation and oxidative stress [17,18,19].Within the sports field, the IF approach seems compatible with a more favorable metabolic profile, and this likely contributes to positive variations in body composition (maintenance of fat-free mass and reductions in fat mass) and exercise performance. Furthermore, IF seems to improve insulin resistance, which is a key factor in using an appropriate metabolic energy distribution. However, there is a lack of studies analyzing the effects of different IF protocols on specific parameters of physical performance [20]. Although fasting is tolerable and safe, it is possible that adverse effects may appear, such as headache, feelings of dizziness and moments of hunger, but maintaining good hydration helps to reduce these effects. Furthermore, extendingfasting for too long, beyond 48 h, leads to numerous harmful side effects on health, such as excessive weight loss, anemia, diarrhea, malnutrition, eating disorders, organic damage and impoverishment of the effect of the immune system [21].

The ketogenic diet is characterized by maintaining a carbohydrate intake below 50grams a day or by being no more than 10% of total energy ingested. Previously, this diet was related to the treatment of epilepsy and as a method of losing weight, and is still used, presenting good results [22,23]. Now it has reappeared with a role closer to the sport field due to the interest generated by aerobic athletes in obtaining a vast energy source. Carbohydrates are mainly stored as glycogen in an organism, which make up about 1680kcal. On the other hand, the energy stored as fat is considered almost unlimited because a pound of fat can contain up to 3500kcal, which extends the endurance of the athletes. However, the beneficial effects of the ketogenic diet on athletic performance remain inconclusive [24,25,26]. Furthermore, although the time course for all changes in body function with KD requires systematic research, maximal changes to muscle fat metabolism occur within 3–4 weeks, and probably 5–10 days of adaptation [10].

The current literature is limited when showing the use of nutritional strategies in the improvement of athletes’ performance, which is usually focused on isolated nutritional strategies. The different and current strategies that are used as key factors in improving the quality of life and performance of athletes, as well as the point up to which the improvements occur and the specific exercise is used, remain unclear. Thus, these gaps need to be addressed as this information is of great importance. This systematic review serves as the basis for future research on the effects of using these dietary strategies in the sports field, in both health and performance optimization, as well as in the prevention of potential injuries.

The main objective of this study is to analyze the scientific evidence on the effectiveness of different nutritional strategies in improving the performance of athletes and their health, and additionally to assess the methodological quality of the selected clinical trials.

## 2. Materials and Methods

### 2.1. Study Design

The present study is a systematic review of the literature of both randomized and non-randomized clinical trials, and was conducted in accordance with the Preferred Reporting Items for Systematic Reviews and Meta-Analyses (PRISMA) statement [27].

### 2.2. Search Strategy

A search for randomized clinical trials according to PRISMA’s methodological criteria for systematic reviews and meta-analyses was carried out [28]. Uncontrolled trials were also added to the search.

Our research question was based on the following elements, following the description of the components of the PECO strategy. (Participants: healthy adult athletes aged between 18 and 65 years; exposure: improvement in the quality of life and optimization of sports performance after application of resistance and strength training; comparator: application or not of alternative nutritional strategies; outcome: effectiveness of nutritional strategies in the health of the athlete and, therefore, in their sports performance; study design: systematic review.)

Two independent investigators (JJPM and MCC) searched the following electronic databases from inception to September 2021 using optimized search strategies: Pubmed, Web of Science, PEDro, Dialnet, Scopus, CINAHL, ProQuest, Medline and Cochrane.

A sensitive search strategy using relevant search terms that were developed from Medical Subject Headings (MeSH) and Descriptors in Health Sciences dictionaries (DeCS) was used. These terms MeSH and DeCS were: “Caloric Restriction” [Mesh], “Sport”, “Fasting” [Mesh], “Strength Training”, “Physical Activity”, “High Intensity Training”, “Resistance Training” [Mesh], “Resistance Exercise”, “Aerobic Exercise”, “Dietary Intervention”, “Dietary Intake” and “Healthy”.

The Boolean operators used were: AND/OR.

The search strategy used in ProQuest and CINAHL was: (“strength training” OR “resistance training” OR “resistance exercise” OR “aerobic exercise” OR “physical activity” OR “sport”) AND (“fasting” OR “caloric restriction”) AND (“healthy”).

Limits and filters applied in ProQuest were: articles, published in the last 5 years, human subjects, age of participants between 19 and 64 years old and language in English or Spanish.

Limits and filters applied in CINAHL were: published in the last 5 years, human subjects, age of participants between 19 and 64 years old and language in English or Spanish.

The search strategy used in Scopus, Medline and Cochrane was: ((“strength training”) OR (“resistance training”) OR (“resistance exercise”) OR (“aerobic exercise”) OR (“physical activity”) OR (sport)) AND ((fasting) OR (“caloric restriction”) OR (“ketogenic diet”)) AND (healthy).

Limits and filters applied in Scopus were: articles, published in the last 5 years and language in English or Spanish.

Limits and filters applied in Medline were: published in the last 5 years, age of participants between 19 and 64 years old and language in English or Spanish.

Limits and filters applied in Cochrane were: clinical trials, published in the last 5 years and language in English or Spanish.

The search strategies used in Pubmed, Web of Science, PEDro and Dialnet were:

Pubmed: “caloric restriction” [Mesh] AND “sports” [Mesh], “fasting” [Mesh] AND “sport” [Mesh], “fasting” [Mesh] OR “caloric restriction” [Mesh] AND sport” [Mesh].

Web of Science: (“fasting” AND sport), (“intermittent fasting” AND sport), (“caloric restriction” AND sport), ((“fasting” OR “caloric restriction”) AND sport).

PEDro: “fasting” AND “sport”.

Dialnet: “intermittent fasting” AND “sport”, “caloric restriction” AND “sport”, “fasting AND sport”.

Limits and filters applied in PubMed were: clinical trial, published in the last 7 years, human subjects, age of participants between 19 and 64 years old and language in English or Spanish.

Limits and filters applied in Web of Science were: clinical trials, published in the last 7 years, human subjects and language in English or Spanish.

Limits and filters applied in PEDro were: clinical trials and published in the last 7 years.

Limits and filters applied in Dialnet were: articles, published in the last 7 years and language in English or Spanish.

### 2.3. Elegibility Criteria

The PECOS framework, as aforementioned, was followed to determine which studies were included in the present systematic review. Each study had to meet the following inclusion criteria: (i) studies that test the effectiveness of fasting on athletes’ performance; (ii) articles should be randomized clinical trials (RCTs) or uncontrolled clinical trials; (iii) human studies published fewer than 7years ago. The exclusion criteria were as follows: (i) studies with subjects with pathologies and (ii) the language of the articles was not English or Spanish.

### 2.4. Study Selection

All studies identified by the search strategy were screened using the eligibility criteria that were specified previously. The first stage of assessment involved the screening of titles and abstracts by two reviewers (JJPM and MCV). The same reviewers undertook the second stage, screening the full text. In cases of disagreement, a decision was made by consensus or, when necessary, a third reviewer (SNL) was consulted.

### 2.5. Data Extraction

Two independent reviewers (JJPM and MCC) who were blinded to each other extracted the following relevant data from each study: study details (first author, year of publication), characteristics of participants, setting, pain condition, SE measuring instrument, outcome measures, duration of follow-up, and study design. If there was any discrepancy between reviewers, a third reviewer was consulted (SNL).

### 2.6. Assessment of Methodological Quality

The evaluation of the methodological quality of the chosen studies was carried out by means of the PEDro scale translated into Spanish [29]. The scale scores 11 items: specified eligibility criteria, random allocation, concealed allocation, similarity at baseline, subject blinding, therapist blinding, assessor blinding, >85% follow up for at least one key outcome, intention-to-treat analysis, between-group statistical comparison for at least one key outcome, and point and variability measures for at least one key outcome. The methodological criteria were classified as follows: if the criterion is met, it obtains 1 point, and if not, 0 points. The first item of the PEDro scale does not count in the score of methodological quality; hence, it was eliminated from the tables to avoid confusion. The PEDro Scale is recognized as a valid measure of the methodological quality of clinical trials due to the findings supporting its use in this category of trials [30]. The “Standard Quality Assessment Criteria for Evaluating Primary Research Papers from a Variety of Fields”(QUALSYT) checklist was used for the quality assessment of non-randomized trials. The scale is made up of 14 criteria that can be answered by four options: Yes, No, Partial and N/A. These items assess whether: the question or objective is sufficiently described, the design is evident and appropriate to answer the study question, the method of subject selection is described and appropriate, the subjects are sufficiently described, if random allocation to treatment group was possible, it is described, if interventional and blinding of investigators to intervention was possible, it is reported, if interventional and blinding of subjects to intervention were possible, it is reported, the outcome and means of assessment are reported, the sample size is appropriate, the analysis is described and appropriate, some estimate of variance is reported, it is controlled for confounding variables, the results are reported in sufficient detail and the results support the conclusions. The maximum obtainable score is 1. The final score is calculated as follows: Total sum = (number of “Yes” × 2) + (number of “Partial” × 1); Possible sum = 28 − (number of “N/A × 2); Final score: total sum/possible sum = ≤1.

To increase the methodological quality of the systematic review, the internal validity of each study was measured by the PEDro Internal Validity Scale(IVS) [29]. Seven internal validity criteria were collected from the PEDro scale. Criteria 2, 3, 5, 6, 7, 8 and 9 were selected to form the IVS and must be classified as follows: Articles with an IVS between 6 and 7 are considered to have a high methodological quality; articles with an IVS between 4 and 5 are considered to have an average methodological quality; articles with an IVS between 0 and 3 are considered to have a low methodological quality.

## 3. Results

### 3.1. Characteristics of the Selected Studies

#### 3.1.1. Study Characteristics

**Figure 1:**
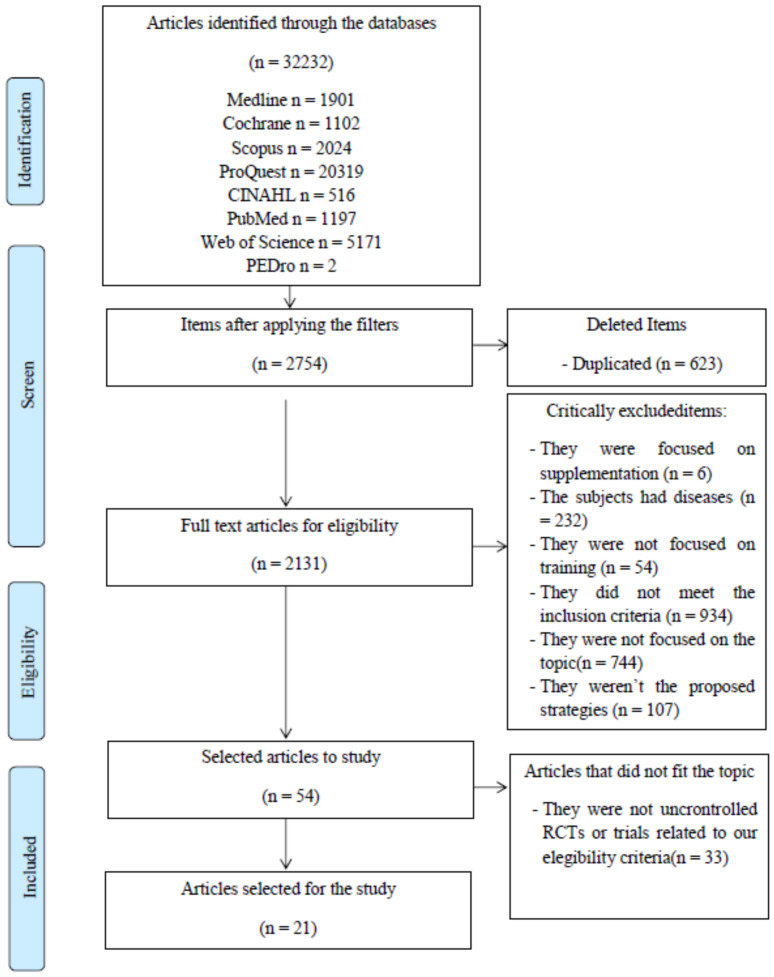
PRISMA screening process for selection of articles for review.

A total of 32,232 citations were identified through electronic databases. The number of studies retrieved from each database and the number of studies excluded in each screening phase are shown in Figure 1. A final number of 21 studies satisfied our inclusion criteria and were included in this review. Of these 21 studies,14 are randomized controlled trials [25,31,32,33,34,35,36,37,38,39,40,41,42,43] and 7 are non-randomized trials [24,44,45,46,47,48,49], with a total of 407 participants who satisfied our inclusion criteria and were included in this review. There are 24 race walkers, 76 cyclist, 21 sprinters, 143 performed resistance training, 12 overload training, 8 runners and 127 participants who did not specify their physical activity.

#### 3.1.2. Methodological Quality

The degree to which studies met the quality criteria varied considerably. The methodological quality assessment of all included studies is presented in Table 1, Table 2, Table 3, Table 4 and Table 5. Characteristics of the selected studies are shown in Table 6.

Seven studies, in addition to being evaluated on the PEDro scale, were also measured on the QUALSYT since they were non-randomized controlled clinical studies.

The characteristics of the intervention and the results of the studies chosen for the review are shown as follows.

### 3.2. Intermittent Fasting

The main differences found between fasting and non-fasting groups were related to physical performance, concentration of glucagon-like-peptide-1, phosphorylation and levels of some proteins related to autophagy (presented LC3I, LC3II and p62 proteins), plasmatic glucose and consumed calories. No differences were found between groups when comparing the systemic concentration levels of the energy-regulating hormones [32].

It is of great importance to specify the results obtained in each kind of exercise given the differences found between them when they are being performed, such as energy production methods and energy distribution, when focusing on aerobic or anaerobic predominance.

In this regard, sprinters decreased speed when they fasted for 14 h per day over a period of 3 days. However, their glucose levels remained stable after training and were higher than those of HDLc and free fatty acids [44]. The concentration of glucagon-like-peptide-1 in the non-fasting group was higher than the fasting group [32].

In endurance sports, the phosphorylation and levels of some proteins related to autophagy have been seen to be higher when fasting for 36 h when compared to untrained subjects [45].

Specific isometric contraction has been measured in sport populations after practicing Ramadan. The levels of voluntary activation and voluntary maximum isometric contraction showed a decrease during the first week of Ramadan, measured by the neuromuscular efficiency and the potential for contraction at rest [46].

Anaerobic and high-intensity performance has been shown to be decreased by assessment with the Wingate and cycling tests after fasting [31].

Finally, fasting before exercise could regulate blood glucose levels, leading to a metabolic shift, which increases fat metabolism and decreases fat storage after a reduction in the total caloric intake [33,50].

### 3.3. Time Restricted Feeding

The mean differences between the TRF groups and the non TRF groups were related to strength, resistance, healthy cardiometabolic markers, decreased body fat, decreased levels of testosterone and insulin growth factor type 1 (IGF-1), increased levels of adiponectin, weight loss, improvements in body composition and an increase in peak power output/bodyweight, and a reduction in white blood cells that usually appear after intense sports. There were no differences in muscle mass loss, hypertrophy, lean mass, muscle measurement in centimeters nor total cholesterol or triglycerides.

Cyclists have been shown to improve body composition and an increase in peak power output/bodyweight (PPO/Body weight) when compared to controls after using TRF intervention [39]. Furthermore, cyclists have shown to improve high intensity aerobic endurance using this nutritional strategy [41].

Strength has been shown to increase after TRF intervention, measured by bench press 1-RM and hip sled 1-RM [35].

Resistance has been shown to increase after TRF intervention, measured by bench press endurance and hip sled endurance [35].

Anaerobic capacity in sprint runners increased in healthy runners when compared to adult runners after a TRF intervention [34].

Health status has been shown to be improved after a TRF intervention, specifically in biomarkers such as blood pressure and increased adiponectin and HDLc, as well as body fat and cardiometabolic markers [37].

### 3.4. Ketogenic Diet

The mean differences between the KD groups and the non-KD groups were related to salivary immunoglobulin A, decreased strength and endurance, decreased stamina, reduction in fat mass and visceral adipose tissue, increased insulin level, an increase in fat metabolism after intense exercise, reduction in body weight by decreasing the fat content and a decrease in muscle damage after exercise. There were no differences between groups when comparing blood pH, concentrations of lactate in the blood and the levels of bicarbonate, VO2max, grip strength and testosterone level.

Resistance trainers have been shown not to improve strength after using a KD intervention when compared to controls [25]. Furthermore, resistance trainers have been shown not to improve endurance after using a KD intervention when compared to controls [25,47]. On the other hand, endurance athletes have reported an improvement in their well-being, easier recovery and benefits in the health of their skin and a reduction in inflammation after using a KD intervention [49].

With regard to muscle strength and hormone profile in male resistance trainers, no significant differences have been found when compared to controls [43].

Cyclists have presented a decrease in the performance of high-intensity exercises, evidenced by a low concentration of lactate after using a KD strategy [48].

## 4. Discussion

The objective of this study is to evaluate the existing evidence on different nutritional strategies in the improvement of the quality of life and performance of the athlete. This review shows that IF and TRF improve the health of the athletes. In terms of performance, there is still controversy in the current research. Aerobic performance, such as resistance or endurance training, seems to be decreased by IF, TRF and KD in the short term, but benefits from nutritional strategies in the long term. Moreover, athletes performing aerobic exercises described an increased well-being, easier recovery, benefits in the health of their skin and a reduction in inflammation after using the KD strategy. Anaerobic exercise, such as strength training, measured by bench press, leg press, lat pull downs and sprints, seem to be increased when using a TRF strategy and decreased when using a KD in the short term. Nevertheless, both strategies may be useful in the long term.

### 4.1. Intermittent Fasting

Physical performance was observed to deteriorate during the first days of the transition period from a usual diet to intermittent fasting in four studies [31,41,44,46]. However, a recovery of performance in the last days of intervention was recognized in two studies [31,41]. In addition, one study [46] explained that intermittent fasting increased the levels of depression, anxiety and fatigue during the first week; hence, the negative effects of IF, which occur at the beginning of this diet, are due to the change in diet. Therefore, a study with longer intervention time may clarify the lack of understanding of intermittent fasting´s effects in the long term.

In the research by Cheriff A et al. [44] and Bin Naharudin MN et al. [31], blood glucose levels were found to remain stable after exercise in fasting subjects. Cheriff A et al. [44] indicate an increase in free fatty acids in blood, while Bin Naharudin MN et al. [31] confirm a decrease in blood triglycerides levels. Thus, a decomposition of triglycerides is observed to create free fatty acids as a source of energy, which causes a decrease in body mass, as explained by Dethlefsen MM et al. [45], without the need to break down muscle tissue.

Dethlefsen MM et al. [45] verified the effects of 36 h of fasting and stated that the levels of phosphorylation and LC3I, LC3II and p62 proteins, related to autophagy, were lower in the non-sports group. In the trained group, autophagy was expected to start between 12 and 24 h after fasting. However, beclin1 was required to start the process, indicating that there were autophagy markers in the blood but not enough to initiate the process in the sports group, which suggests that a 36 h fast may serve to regulate skeletal musculature autophagy in subjects who perform sports. Furthermore, Edinburgh et al. [33] showed that exercise during fasting decreased plasmatic glucose levels by modulating them when compared to controls that carried out exercise after having breakfast.

Three main theories have been suggested to explain the benefits of IF on improving metabolic effects, such as better insulin sensitivity, and on metabolic markers, such as systolic blood pressure, HbA1c, fat mass and triglycerides, even in eucaloric conditions: (i) the Ketosis theory, (ii) oxidative stress hypothesis and (iii) the circadian rhythm hypothesis [19]. The first is widely known for its postulated mechanism of metabolic shift, which increases fat metabolism and decreases fat storage after a reduction in total calorie intake. The second focuses on mitochondrial metabolism, which decreases the cellular inflammatory process. The third is thought to affect our internal 24 h circadian rhythm at both a central and peripheral cellular level when altering the timing of food intake. These theories open new possibilities to plan the specific moment to use a nutritional strategy based on a specific goal. In the performance of athletes, this may be to increase insulin sensitivity or to improve energy metabolism based on the moment of the match or training. However, there is little evidence in this regard and more studies are needed to propose optimal IF protocols for athletes [50].

### 4.2. Time-Restricted Feeding

The TRF has managed to equal [36,38] and even surpass [35,39] the improvements in strength and endurance of the control group by carrying out different diets, but with similar caloric content. This shows that that TRF does not decrease sports performance and can be practiced by athletes without fear of losing performance. Gasmin et al. [34] showed that the use of two days per week of TRF for 3 months increased the level of red and white blood cells, hemoglobin, hematocrit and neutrophiles in young athletes when compared to old athletes, which suggests that the efficacy of this nutritional strategy may be related to age.

With regard to the development of muscle mass, Grant M et al. explains that although there was no decrease in it, the control group achieved a significant increase compared to the intervened group [35]. On the other hand, Grant M et al. and Moro T et al. stated that there was no significant difference in the improvements of the groups [36,38]. The differences between the studies most likely lie in the intervention times, the exercises performed during the intervention and the range of hours in which the subjects were fed. Therefore, it can be concluded that there is no decrease in muscle mass when practicing such a diet.

There was a significant reduction in terms of body fat in the intervened group in three studies [37,38,39]. Hence, the maintenance of lean body mass and decreasing fat could be considered a good method to control body weight.

As regards endocrine effects, it should be noted that in the three aforementioned studies adiponectin was observed, which accompanies an improvement in insulin affinity [37,38,39]. Both Moro T et al. and Moro T et al. reflected a decrease in testosterone and IGF-1 levels after exercising in the intervened group [38,39]. However, Moro T et al. indicated that white blood cell levels decreased significantly less in the TRF group than in the control group after intense exercise [39].

McAllister MJ et al. indicates a reduction in blood pressure and increased HDLc in the intervened group [37]. On the other hand, Moro T et al. states that there was no significant variation in cholesterol levels in any group. The difference in outcomes in both articles probably comes from the different exercises and intervention times applied in each study [38].

In the study by Grant M et al., the effects of TRF were verified along with HMB supplementation, but did not show significantly different results compared to the group that only practiced TRF [36].

### 4.3. Ketogenic Diet

This diet focused on fat metabolism as a source of energy and is supported by two studies. Two studies agree that there was an increase in fat metabolism significantly in the subjects who followed this diet [48,49]; however, other studies [24,25,47] did not show a significant difference between the control and the intervened group. The differences in outcomes may be due to the distinct characteristics of the individual subjects recruited. Carr AJ et al. indicated that among the three groups that participated in the study, there were no significant differences between groups, arguing that this could be caused by the high performance capacity of the subjects, who were elite athletes [24].

Research in three studies showed that body mass was reduced in the intervened group [25,48,49]. However, Kysel C et al. and Gasmin et al. indicate that in addition to reducing fat content, lean body mass was also metabolized [25,34].

The studies of Kysel P et al. [25], Zajac A et al. [48] and Zinn C et al. [49] stated that the participants felt that their athletic performance was significantly reduced compared to the controls and therefore they did not consider it a good diet to improve physical performance. Specifically, Sjodin et al. [40] concluded that a ketogenic diet decreased endurance in physically active women while they were using said nutritional strategy.

On the other hand, the participants of Zinn C et al. [49] indicated an improvement in their well-being, greater speed of recovery, benefits in the health of their skin and a reduction in inflammation.

Our results are in line with current research on the effects of KD in the athletic population. It affects physical health and has positive effects on fat oxidation but shows conflicting results regarding the effects of a KD on performance, which are mostly shown in the long term. Thus, there are both beneficial and detrimental effects after using a KD strategy in athletic populations [10,51,52,53]. Further research is required to establish recommended protocols regarding a KD for athletes. Furthermore, deleterious effects on stool microbiota and iron metabolism have been shown [51].

Further research exploration is still needed using different intervention times and variation in the individual characteristics of subjects, with different types of exercise and sports levels.

Finally, current research shows limited evidence when sub-grouping findings to specific athletic populations when using nutritional strategies to improve performance. In this regard, in order to compound the findings, the effects of specific nutritional interventions to improve performance has only been possible to be shown in relation to resistance athletes, sprinters and cyclists.

Resistance athletes have been shown to increase performance when using IF and TRF interventions, but not when using KD. However, long-term benefits have been shown after using a KD intervention.

Professional sprinters have been shown to increase performance when using TRF but decrease it after using an IF intervention.

Professional cyclists have shown to increase performance when a TRF intervention has been followed.

### 4.4. Strengths and Limitations of the Study

First, the methodological quality of the literature was reviewed with the PEDro, IVS and QUALSYT scales, which increases the quality of the present study and consequently the quality of the results obtained. Second, the review of the literature deals with an innovative topic in which eating habits in the field of sports and the aim of improving the health and performance of the athlete are combined. Third, the conclusions of the review serve as a proposal for future studies in which variations in the health and performance of athletes are analyzed after the proposed interventions.

However, some limitations have to be recognized. Some variables in the results may be produced by the individual characteristics of the subjects, for example the different sports performed which included race walkers, cyclists, sprinters, runners, resistance training and overload training, and, furthermore, there were 127 participants who did not specify their preferred physical activity. In addition, because of heterogeneity and risk of bias between studies, we could not reach a clear consensus about specific effects of IF, TRF and KD on specific aerobic or anaerobic sports or sub-grouping. Interventions were based on changing the eating habits of sports subjects; hence, participation was limited. Additionally, the length of the intervention studies was also limited. Finally, this review concluded that the analyzed articles presented a medium level, a low level and a high level of methodological quality in the PEDro scale, the IVS scale and the QUALSYT scale, respectively. Thus, the results from the present review should be interpreted with caution. Apart from PEDro, IVS and QUASYLT used in this study, grey literature databases such as NHS Evidence, New York Academy of Medicine Grey Literature Report, Explore the British Library, TRIP database, National Guideline Clearinghouse, Grey Source, and Open Grey may be explored to detect any relevant unpublished work. However, due to the huge number of references obtained in the main search we considered it unnecessary to increase the search with unpublished work.

### 4.5. Clinical Application of the Results

Intermittent fasting and time-restricted feeding allow athletes to maintain their physical performance in the same way as with their usual diets, but with the presence of numerous positive effects, such as body weight control by metabolizing fat as an energy source without breaking down muscle tissue, increasing adiponectin levels and consequently improving insulin sensitivity, maintaining white blood cell levels after intense exercise, reducing systemic inflammation caused by low levels of glucose and omega 6 and finally improving personal well-being. However, these nutritional strategies are not in line with recommendations from international organizations such as the International Olympic Committee, the Academy of Nutrition and Dietetics, the American College of Sports Medicine, and Dieticians of Canada, which indicate that athletes with high training loads should consume a high-carbohydrate diet [9].

These effects can be effective in preventing injuries and optimizing healing processes under clinical conditions. By reducing systemic inflammation, controlling body weight, improving insulin sensitivity, and strengthening the immune system, health and quality of life greatly improve. Furthermore, these health benefits will prevent injuries, such as muscle inflammation caused by the accumulation of lactic and pyruvic acid, joint wear or muscle tear, as well as the acceleration of the healing process resulting in optimal recovery. In this regard, IF before exercise may be used as an injury prevention strategy. A recent study by Navarro et al. showed significant differences in load tendon thicknesses (patellar tendon and Achilles tendon) between runners with different number of meals per day, as well as a negative association between tendon thicknesses and meals per day. The hypothesis behind this is based on the likely insulin resistance and hyper-insulinemia state in the athlete, which induces the overexpression of undercarboxylated osteocalcin and loss of bone density and collagen tissue damage by activation of RANKL in bones and increased uptake of glucose in collagen tissue, respectively. The increase in intracellular glucose activates the polyol pathway that induces the production of sorbitol and fructose under severe oxidative stress, which could be responsible for mitochondrial damage and tissue breakdown [54].

On the other hand, although it is out with the focus of the study, it is important to highlight that current research shows nutritional interventions as strategies that benefit the nervous system. In this context, IF produces effects through metabolic, cellular, and circadian mechanisms, leading to anatomical and functional changes in the brain, which might be protective in the development of neurological disorders. Theoretically IF may also be beneficial for neurodevelopmental and mood disorders, but hardly any experimental data exists on this topic [55,56]. Nutritional strategies in order to improve both central and peripheral nervous system may be of great interest to increase health and performance in athletes and future studies are needed [57].

### 4.6. Future Studies

Studies analyzing the effects of different nutritional strategies, not only IF, TRF or KD but others such as the Mediterranean and vegan diets, on performance, quality of life, nervous system and brain improvement, prevalence of injuries and injury recovery times in athletes, not only in the short but also in the long term, are necessary, as well as specific data on both aerobic and anaerobic sports.

IF before exercise may be used as an injury prevention strategy, but more studies in this line are needed.

Furthermore, studies taking the circadian rhythm into account when planning the timing of food intake based on the time of a match or training in order to increase the athlete’s performance would be of great interest.

## 5. Conclusions

The methodological quality of the studies assessed with the PEDro scale is considered medium, the internal validity of them is considered low and those valued with the QUALSYT scale had a high quality.

TRF may improve athletic performance in both professional sprinters and resistance athletes through increasing adiponectin levels and insulin sensitivity and decreasing both testosterone and IGF-1 levels after intense physical activity.

An athlete undertaking 5–6 h of exercise after intermittent fasting will improve their performance, specifically resistance athletes, although inconclusive results exist for anaerobic exercise where it may be impaired within the first week.

Both IF and TRF decrease body mass without significantly affecting lean mass.

The ketogenic diet reduces body mass by consuming fat, but with the risk of catabolizing lean mass. In relation to performance, controversy still exists in the current research, since there are both beneficial and detrimental effects after using a KD strategy in athletic populations.

The use of different dietary strategies may improve the health and performance of athletes, mainly in the long term after using a KD, while IF and TRF may improve performance in professional sprinters and resistance athletes in the short term.

## Figures and Tables

**Table 1:** Methodological assessment with PEDro scale. Articles 1 to 10.

Item	BinNaharudin et al. (2018)	Carr, et al. (2018)	Cheriff et al. (2016)	Chowdhury et al. (2018)	Dethlefsen et al. (2018)	Edinburgh et al. (2019)	Gasmi et al. (2017)	Grant et al. (2017)	Grant et al. (2019)	Gueldich et al. (2019)
**2**	X			X		X	X	X	X	
**3**									X	
**4**		X		X		X	X	X	X	
**5**										
**6**									X	
**7**				X						
**8**	X	X	X			X	X			X
**9**	X		X			X			X	X
**10**	X	X	X	X	X	X	X	X	X	
**11**	X	X	X	X	X	X	X	X	X	X
**TotalPEDro**	**5/10**	**4/10**	**4/10**	**5/10**	**2/10**	**6/10**	**6/10**	**4/10**	**7/10**	**3/10**

1. The selection criteria were specified. 2. Subjects were randomly assigned to groups. 3. The assignment was hidden. 4. The groups were similar at baseline in relation to the most important prognostic indicators. 5. All subjects were blinded. 6. All therapists who administered the therapy were blinded. 7. All assessors who measured at least one key outcome were blinded. 8. Measures of at least one of the key outcomes were obtained from more than 85% of the subjects initially assigned to the groups. 9. Results were presented for all subjects who received treatment or were assigned to the control group, or where this could not be the case, the data for at least one key outcome were analyzed by ‘intention to treat’. 10. Results of statistical comparisons between groups were reported for at least one key outcome. 11. The study provides point and variability measures for at least one key outcome. Different colors represent the methodological quality of studies with a high (red), or unclear (yellow) or low (green) risk of bias and applicability concerns.

**Table 2:** Methodological assessment with PEDro scale. Articles 11 to 21.

Item	Kysel et al. (2020)	McAllister et al. (2019)	Moro et al. (2016)	Moro et al. (2020)	Shaw et al. (2020)	Sjödin et al. (2020)	Terada et al. (2019)	Vargas et al. (2018)	Vidic’ et al. (2021)	Zajac et al. (2014)	Zinn et al. (2017)
**2**	X	X	X	X	X	X	X	X	X	X	
**3**											
**4**	X	X	X	X		X	X	X	X	X	
**5**											
**6**	X										
**7**			X	X		X	X				
**8**	X	X	X	X			X	X	X	X	
**9**	X	X	X	X			X	X		X	
**10**	X	X	X	X	X		X	X	X	X	
**11**	X	X	X	X	X	X	X	X	X	X	X
**TotalPEDro**	**7/10**	**6/10**	**7/10**	**7/10**	**3/10**	**4/10**	**7/10**	**6/10**	**5/10**	**6/10**	**1/10**

1. The selection criteria were specified. 2. Subjects were randomly assigned to groups. 3. The assignment was hidden. 4. The groups were similar at baseline in relation to the most important prognostic indicators. 5. All subjects were blinded. 6. All therapists who administered the therapy were blinded. 7. All assessors who measured at least one key outcome were blinded. 8. Measures of at least one of the key outcomes were obtained from more than 85% of the subjects initially assigned to the groups. 9. Results were presented for all subjects who received treatment or were assigned to the control group, or where this could not be the case, the data for at least one key outcome were analyzed by ‘intention to treat’. 10. Results of statistical comparisons between groups were reported for at least one key outcome. 11. The study provides point and variability measures for at least one key outcome. Different colors represent the methodological quality of studies with a high (red), or unclear (yellow) or low (green) risk of bias and applicability concerns.

**Table 3:** Assessment of internal quality using IVS scale. Articles 1 to 10.

Item	BinNaharudin, et al. (2018)	Carr et al. (2018)	Cheriff et al. (2016)	Chowdhury et al. (2018)	Dethlefsen et al. (2018)	Edinburgh et al. (2019)	Gasmi et al. (2017)	Grant et al. (2017)	Grant et al. (2019)	Gueldich et al. (2019)
**2**	X			X		X	X	X	X	
**3**									X	
**5**										
**6**									X	
**7**										
**8**	X	X	X	X		X	X			X
**9**	X		X			X			X	X
**IVS**	**3/7**	**1/7**	**2/7**	**2/7**	**0/7**	**3/7**	**2/7**	**1/7**	**4/7**	**2/7**
**Quality**	**Low**	**Low**	**Low**	**Low**	**Low**	**Low**	**Low**	**Low**	**Medium**	**Low**

Different colors represent the methodological quality of studies with a high (red), or unclear (yellow) or low (green) risk of bias and applicability concerns.

**Table 4:** Assessment of internal quality using IVS scale. Articles 11 to 21.

Item	Kysel et al. (2020)	McAllister et al. (2019)	Moro et al. (2016)	Moro et al. (2020)	Shaw et al. (2020)	Sjödin et al. (2020)	Terada et al. (2019)	Vargas et al. (2018)	Vidic´ et al. (2021)	Zajac et al. (2014)	Zinn et al. (2017)
**2**	X	X	X	X	X	X	X	X	X	X	
**3**											
**5**											
**6**	X										
**7**			X	X		X	X				
**8**	X	X	X	X			X	X	X	X	
**9**	X	X	X	X			X	X		X	
**IVS**	**4/7**	**3/7**	**4/7**	**4/7**	**1/7**	**2/7**	**4/7**	**3/7**	**2/7**	**3/7**	**0/7**
**Quality**	**Medium**	**Low**	**Medium**	**Medium**	**Low**	**Low**	**Medium**	**Low**	**Low**	**Low**	**Low**

Different colors represent the methodological quality of studies with a high (red), or unclear (yellow) or low (green) risk of bias and applicability concerns.

**Table 5:** Methodological assessment using the QUALSYT.

ITEM	Carr et al. (2018)	Cheriff et al. (2016)	Dethlefsen et al. (2018)	Gueldich et al. (2019)	Shaw et al. (2020)	Zajac et al. (2014)	Zinn et al. (2017)
**1**	Yes	Yes	Partial	Partial	Yes	Yes	Parcial
**2**	Yes	Yes	Yes	Yes	Yes	Yes	Partial
**3**	Partial	Yes	Yes	Partial	Yes	Partial	Partial
**4**	Yes	Partial	No	Yes	No	Yes	Yes
**5**	No	N/A	N/A	N/A	N/A	N/A	N/A
**6**	No	N/A	N/A	N/A	N/A	N/A	N/A
**7**	No	N/A	N/A	N/A	N/A	N/A	N/A
**8**	Yes	Yes	Yes	Yes	Yes	Yes	Yes
**9**	Yes	Yes	Yes	Yes	Yes	Yes	Partial
**10**	Yes	Yes	Yes	Yes	Yes	Yes	Yes
**11**	Yes	Yes	Yes	Yes	Yes	Yes	Partial
**12**	Yes	Partial	Partial	Yes	Yes	Yes	N/A
**13**	Yes	Yes	Yes	Yes	Yes	Yes	Yes
**14**	Yes	Yes	Yes	Yes	Yes	Yes	Yes
**Total**	**0.75**	**0.90**	**0.81**	**0.90**	**0.90**	**0.95**	**0.75**

1. The purpose is specifically explained. 2 The population studied was clearly explained. 3. The participation rate for eligible individuals was at least 50%. 4. All subjects were recruited from the same or similar population. 5. The sample size, a description or estimate of the variance and effect were justified. 6. Exposures of interest were measured before the results were performed. 7. The time period was long enough to reasonably expect to see an association between the exposure and the outcome. 8. The study was examined at different exposure levels with respect to the outcome. 9. Exposure measures were clearly defined, valid, reliable, and consistently implemented in the participants. 10. Exposures were evaluated more than once over time. 11. Outcome measures were clearly defined, valid, reliable, and consistently implemented in the participants. 12. Outcome assessors were blinded to the exposure status of the participants. 13. Loss to follow-up after the start of the study was less than 20%. 14. Key confounders were measured and statistically adjusted for their impact on the exposure–outcome relationship. Different colors represent the methodological quality of studies with a high (red), or unclear (yellow) or low (green) risk of bias and applicability concerns.

**Table 6:** Characteristics of the selected studies.

Name	Type of Study	Sample	Intervention	Measurements	Results
BinNaharudin et al. (2018)	RCT	N = 20 men Control: 10 Age: 20 ± 1 Intervention: 10 Age: 21 ± 1 Losses: 0	Both groups had a trial period to get used to the program for 7 days. Every 2 days, they performed the Wingate Test and in the next session, a prolonged high-intensity cycling test, both in a period of 10 days. This period was repeated twice interspersing a 4-week break. The control 5 times a day consumed a total of 2492 ± 20 kcal. The intervention 4 times a day consumed a total of 1500 ± 55 kcal by skipping lunch.	VO2 peak. Blood and urine samples. Body mass.	From day 4 the intervention decreased their body mass (*p* < 0.001). Decrease in the performance of the Wingate Test in the intervention during the first days (*p* < 0.05). However, it recovered after the fourth day (*p* < 0.05). Decrease in the performance of the cycling test in the intervention during the sessions, but there was a tendency to recover performance in the later phases. Compared to Day 0, the cycling test performance in the intervention group reduced on Day 2 (*p* < 0.0001), Day 4 (*p* < 0.001), Day 6 (*p* < 0.001), Day 8 (*p* < 0.05) and Day 10 (*p* < 0.05). After the exercises, the intervention had higher blood glucose levels (*p* < 0.05) and lower blood levels of triglycerides in the last sessions (*p* < 0.01).
Carr et al. (2018)	Non-randomized parallel group study	N = 28 (17 men and 7 women) Control: 8 Intervention A: 9 Intervention B: 7 Losses: 4	All groups followed an intensive supervised training session every day for 3 weeks. Control had a high-carb diet. Intervention A had a low-carb and high-fat diet. Intervention B had a diet of periodic carbohydrates.	Walking economy. VO2 peak tested on treadmill. Net endogenous acid production (NEAP). Blood samples.	Within each of the three groups, there was a significant increase in VO2max compared with baseline (*p* < 0.05). Blood pH: At 4 min and 6 min post-exercise there were no significant difference between pH of HCHO and LCHF (95% CI = (−0.10; 0.02) and (−0.08; 0.04), respectively. Bicarbonate: there were no significant differences in post-training intervention blood [HCO3-] between groups (F(1,161.15) = 0.14; *p* = 0.71). Lactate: Post-intervention, there were no significant differences between groups (F(14,311.07) = 1.28; *p* = 0.22). Post-intervention, NEAP was significantly higher in LCHF compared with HCHO control (95% CI = (10.44; 36.04)) but there was no difference for PCHO compared with HCHO (95% CI = (−19.06; 7.23)).
Cherif et al. (2016)	Counterbalanced test Quasi-experimental study Uncontrolled trial	N = 21 men Age: 29.8 ± 5.9 Intervention: 21 Losses: 0	In both sessions, endurance tests were carried out based on sprint repetitions. Control session: they followed a normal diet. Intervention session: fasted 14 h for 3 days in a row. After 7 days of intervention, the diets are exchanged between groups.	Biomechanical and biochemical markers measured by blood samples and body mass index.	Speed sprint and vertical stiffness declined in the fasting session compared to the control session (*p* = 0.030). During the two first sprints of the second repeated sprints set, sprint speed significantly decreased in the fasting session (*p* = 0.003) compared to the control session (*p* = 0.048), while horizontal power declined in the fasting session compared to the control session only in sixth sprint (*p* = 0.017). Triglyceride levels improved during fasting (*p* = 0.037). Total cholesterol (*p* = 0.581) and LDL-c (*p* = 0.835) levels did not vary in this session. HDL-C was higher in fasting session compared to control session at post-exercise (*p* = 0.039).
Chowdhury, et al. (2018)	RCT	N = 31 (12 men and 19 women) Control: 15 (6 men and 9 women) Age: 36 ± 12 Intervention: 16 (6 men and 10 women) Age: 35 ± 10 Losses: 0	For 6 weeks: Control group followed a diet which consisted of ingesting ≥ 700 kcal, during the first 2 h after waking up, of the 1100 kcal daily maximum Intervention group remained in fasted state until midday. Their total daily intake was 1200 kcal.	Body mass Resting metabolic rate Appetite Score	There were no differences between the levels of energy regulation, glycerin, peptides and appetite. Glucagon concentrations were lower in the intervention group compared to control group, where it went up (*p* = 0.06).
Dethlefsen et al. (2018)	Quasi-experimental study Uncontrolled trial	N = 17 men Trained: 6 Age: 27 ± 4 Untrained: 7 Age: 28 ± 3 Losses: 4	Both groups fasted for 36 h and samples were taken 2, 12, 24 and 36 h after the last meal. The trained group had a VO2max above 55 mL·min^−1^·kg^−1^. The untrained group had a VO2max below 45 mL·min^−1^·kg^−1^.	Amino acid quantification. Lysing of muscle tissue. Polyacrylamide gel on electrophoresis with sodium dodecylsulfate followed by Western blotting. Blood samples.	Phosphorylation and protein levels of several proteins related to autophagy were higher in the trained group (*p* < 0.05). Skeletal muscle LC3I protein content was ~30% lower (*p* < 0.05) at 12, 24, and 36 h than at 2 h after the meal in untrained subjects. The LC3II protein content in skeletal muscle was ~20% lower (*p* < 0.05) at 12 and 24 h and tended to be lower (*p* = 0.059) at 36 h than at 2 h after the meal in untrained subjects. The p62 protein content in skeletal muscle was ~60% lower (*p* < 0.05) at 24 h than 2 h after the meal in untrained subjects, whereas fasting had no effect on p62 protein content in trained subjects.
Edinburgh et al. (2019)	RCT	N = 12 men Age: 23 ± 3 Intervention: 12 Losses: 0 1.1.1	Subjects followed 3 different programs for a 24 h period with 1 week of washout between them: Breakfast followed by rest: breakfast was eaten and no type of exercise was performed. Breakfast before exercise: breakfast was eaten and 60 min of cycling at 50% peak power output was performed. Fasting before exercise: breakfast was skipped and 60 min of cycling at 50% of peak power output was performed.	Blood samples Expired gas samples Energy expenditure Energy intake	Participants who fasted before exercise strategy consumed less calories compared to the other strategies (*p* < 0.01). Energy expenditure was only significatively lower when breakfast was followed by rest (*p* < 0.01). There was a significant usage of plasmatic glucose with the fasting before exercise strategy (*p* = 0.03).
Gasmi et al. (2017)	RCT	N = 40 men Control: 20 Young men: 10 Age: 24.90 ± 1.10 Aged men: 53.90 ± 4.09 Intervention: 20 Young men: 10 Age: 26.90 ± 1.97 Aged men: 10 Age: 51.60 ± 5.87 Losses: 0	During the months February, March and April: Intervention group followed 12 h of fasting 2 days a week with a rest stage of 48 h (Monday and Thursday) Control group kept their usual diet.	Energy (kcal/day) Fat Wmax Red cells Hemoglobin Running-based anaerobic sprint test	There was no significant difference in muscular strength in any group. Concentration levels of hemoglobin, red cells and white cells were significantly higher in young participants (*p* > 0.05). There was no improvement in levels of CD3, CD4+, and CD8+ (*p* > 0.05).
Grant et al. (2017)	RCT	N = 28 men Control: 8 Age: 22.0 ± 2.4 Intervention: 10 Age: 22.9 ± 4.1 Losses: 10	Both groups performed a resistance training program 3 days/week for 8 weeks. Control continued with his usual diet. Intervention could only eat in a period of 4 h a day 4 times a week without limitation of quantity.	Body composition. Muscle performance.	The values of body composition were lean soft tissue (*p* = 0.30), fat mass (*p* = 0.14) and body fat (*p* = 0.37). Muscular performance increased in both groups, but the intervention group showed greater improvements in lower body strength and upper and lower body endurance. The exercises that evaluated the muscular performance were bench press 1-RM (*p* = 0.35), bench press endurance (*p* = 0.17), hip sled 1-RM (*p* = 0.07) and hip sled endurance (*p* = 0.97). There was greater increase in lean soft tissue in the control group of 2.3 Kg on average, as opposed to −0.2 Kg in intervention group, but it is not a significative difference.
Grant et al. (2019)	RCT	N = 40 women Control: 14 Age: 22.0 ± 2.4 Intervention A: 13 Age: 22.1 ± 2.1 Intervention B:13 Age:22.3 ± 3.4 Losses: 16	All groups completed an endurance training program for 8 weeks. Control followed a normal diet. Intervention A: could consume calories between 12 am and 8 pm hours. Intervention B: could consume calories between 12 am and 8 pm in addition to consuming β-hydroxy β-methyl butyrate supplements.	Lean mass and fat rates. Body composition. Muscle performance. Resting metabolic rate and use of substrates. Brachial blood pressure. Blood and saliva samples.	Fat rate: statistically significant changes in favor of intervention (*p* = 0.12). Muscular performance increased in all groups with no significant differences between them. The analyzed values were resistance training volume (*p* > 0.05), muscular performance (*p* > 0.05), rate of force development (*p* > 0.05) vertical jump performance (*p* > 0.05), physical activity energy expenditure (*p* = 0.034), sedentary time (*p* = 0.048), moderate- or vigorous-intensity physical activity (*p* > 0.05), steps per day (*p* > 0.05). There are no significant differences in metabolic and physiological characteristics. The analyzed values were Urinary HMB concentrations (*p* < 0.01), body composition (*p* > 0.05), resting metabolism (*p* > 0.05), blood variables (*p* > 0.05), vascular assessments (*p* > 0.05) and cortisol awakening response (*p* > 0.05).
Gueldich et al. (2019)	Quasi-experimental study Uncontrolled trial	N = 10 men Age: 22.06 ± 1.98 Intervention: 10 Losses: 0	The intervention fasted for a month following the tradition of Ramadan. The group performed 3 repetitions of a maximum voluntary isometric contraction of knee extension with electrostimulation during 4 phases: one week before Ramadan, at the end of the first week, during the fourth week and two weeks after its completion	Voluntary activation level (VAL). Electromyographic signals. Contraction potential at rest. Depression, anxiety and fatigue: Profile of Mood States questionnaire in French version (POMS-f). Maximum voluntary isometric contraction (MVIC) values. 1.1.2	VAL (*p* < 0.05) and MVIC (*p* < 0.001) values decreased during the first week of Ramadan. Neuromuscular efficiency (*p* = 0.15) and the potential of contraction at rest (*p* = 0.07) remained stable throughout the month. The values of the POMS-f were higher during the first week. Significant difference in comparison with before Ramadan and the other measured days. *p* < 0.005 in week 1, *p* < 0.01 in week 4 and *p* < 0.001 after Ramadan.
Kysel et al. (2020)	RCT	N = 25 men Control: 12 Age: 24 ± 4 Intervention: 13 Age: 23 ± 5 Losses: 0	During an 8-weekperiod, both groups followed a programmed diet with strength and resistance training. Muscles from chest, back and legs were trained in 3 sessions on 3 different days. Resistance training consisted of a 30 min run at constant heart rate at 70% of maximal heart rate. In addition, the total energy intake of each participant was calculated and reduced by 500kcal per day. Cyclical ketogenic reduction diet: first 5 days of the week the carbohydrate consumption was reduced to 30 g per day and on the last 2 days it was increased to 8–10 g per kg of non-fat tissue. Nutritionally balanced reduction diet: only the 500 kcal per day restriction was included.	Body composition Muscle strength Endurance performance Peak workload Peak oxygen uptake	Both groups decreased body weight, body fat mass and body mass index. Lean body mass and body water content were significantly reduced by the intervention, while they were not influenced in control group (*p* < 0.05). Intervention did not produce a significant improvement in muscle strength, while control group was able to improve it (*p* < 0.05). Respiratory exchange ratio decreased in subjects on intervention while it did not vary in control group (*p* < 0.05). Spiroergometric results were higher in control group (*p* < 0.05).
McAllister et al. (2019)	Pilot study Randomized and controlled	N = 26 men Age: 22 ± 2.5 Control: 12 Intervention: 10 Losses: 4	Both groups could only eat in a period of 8 h a day for 28 days. The control (ab libitum) could consume as much as they wanted in the set time interval. The intervention (isocaloric) should be kept 300 kcal/day below a normal daily intake.	Blood pressure. Body composition. Blood samples. Hunger, satiety, concentration, mood, energy, alertness and focus: visual analogue scale.	In both groups, body fat and blood pressure decreased (*p* < 0.05). In both groups there were increases in HDL-c and adiponectin (*p* < 0.05).
Moro et al. (2016)	RCT	N = 34 men Age: 29.21 ± 3.8 Control: 17 Intervention: 17 Losses: 0	Both trained for 8 weeks (3 sessions per week) in a resistance training program. Control: ate at 8 am, 1 pm and 8 pm. Intervention: ate at 1 pm, 4 pm and 8 pm.	Height and weight. Fat and lean mass rates. Muscle areas Aspirated oxygen and expelled carbon dioxide. Blood samples. Upper and lower limb strength.	Greater decrease in fat (*p* = 0.0448) and maintenance of lean mass in favor of intervention group. Increased strength in both groups without a significant difference between them ( *p* > 0.05). Insulin growth factor levels type 1 (*p* = 0.0397) and testosterone (*p* = 0.0476) decreased in intervention group. Decreased respiratory rate in intervention group (*p* = 0.0421). Decreased glucose (*p* = 0.0011) and insulin levels (0.0303) in intervention group. Due to that, there were an improvement in the homeostatic model assessment in intervention group.
Moro et al. (2020)	RCT	N = 16 men Control: 8 Age: 19.38 ± 1.60 Intervention: 8 Age: 19.38 ± 2.39 Losses: 0	Both groups cycled 500 ± 50 km a week divided into 6 weekly cycling sessions for 4 weeks. Controls (ND) consumed a complete diet divided into 3 meals between 7 am and 9 pm. Intervention (ERT) consumed a complete diet at an interval of 8 h (10 am to 6 pm).	Body composition. Resting metabolic rate. Peak power output (PPO). Blood samples.	Reduction inbody fat in favor of the intervention group (*p* = 0.01). The resting metabolic rate has no significant interaction between groups (*p* > 0.05). There is no significant difference between groups in sports performance (*p* > 0.05). The intervention shows a decrease in inflammatory markers (*p* < 0.005). Leucocytes decreased in both groups (*p* = 0.001), but the difference between baseline and final values was only significant for the control group.
Shaw et al. (2020)	Cross design Uncontrolled trial	N = 10 men Intervention: 8 Age: 29.6 ± 5.1 Losses: 2	For 31 days, participants were on a certain diet with a week of interspersed rest. The intervention with ketogenic diet consumed <50 g/day of carbohydrates. Diet was composed of 15–20% proteins and 80–85% fats. The intervention with his usual diet did not vary their consumption.	Blood and saliva samples. Incremental exercise test to exhaustion: days 1 and 31 ran to exhaustion at 70% of their VO2 peak.	Diets had no significant effects on VO2 peak or exercise tests until exhaustion (*p* > 0.05). The ketogenic diet can alter the pro- and anti-inflammatory immune response of cytokines (*p* < 0.05) and the segregation of immunoglobulin A (*p* < 0.001).
Sjödin et al. (2020)	RCT	N = 24 women Age:18–30 Control: 8 Intervention: 9 Losses: 7	During a 4-week period, each group followed their programmed diet. After the first intervention was complete, a washout period of 15 weeks took place. The groups then interchanged their diets for another 4 weeks. Control group followed a program diet from the National Food Agency. Intervention group followed a ketogenic diet.	VO2 max Lactate Handgrip time to fatigue and strength Graded Incremental Ergometer Cycling Test	The first results did not show a significant change in strength and time to fatigue in the intervention group. Time to fatigue in cycling test was reduced by 2 min in intervention group (*p* < 0.001). Participants experienced an improvement in muscular fatigue during the exercise days. Ketogenic diet had an unfavorable effect on muscle fatigue and might affect perceived exertion during daily life activities.
Terada et al. (2019)	RCT	N = 25 men Control: 9 Age: 34.0 ± 8.2 Intervention: 11 Age: 33.3 ± 7.2 Losses: 5	Both groups trained 4 weeks (3 sessions/week). Aerobic cycling of maximum intensity. Control: they consumed exogenous carbohydrates. Intervention: night fasting.	High intensity aerobic endurance: T85%. Aerobic capacity: (VO2 peak). Mechanical work.Peak power output (PPO). Fatigue rate.	Mean of T85% was longer in the intervention (*p* = 0.038). Aerobic capacity did not vary between groups (*p* > 0.05). Mechanical work (*p* = 0.010) and Peak Power Output (*p* = 0.021) were lower in the intervention group, but still can have a greater impact on the ability to sustain high-intensity aerobic endurance exercise compared to control group. Fatigue rate did not vary between groups (*p* > 0.05).
Vargas et al. (2018)	RCT	N = 26 men Age: 30 ± 4.5 Control: 5 Intervention A: 9 Intervention B: 10 Losses: 2	Participants were organized into 3 groups to participate in the 8-week-long study: Control group: they did not follow any programmed diet or training during the study. Intervention A: followed a ketogenic diet Intervention B: followed a non-ketogenic diet The exercise training consisted of 4 sessions per week (1 session/day) and 3 days of rest. Upper and lower limbs were trained separately in 2 sessions for each one.	Body composition	There was significant reduction in fat mass and visceral adipose tissue in the ketogenic diet (*p* < 0.05). Total body weight and muscle mass were significantly higher in the non-ketogenic group compared to the ketogenic group, which stayed neutral (*p* < 0.05).
Vidic et al. (2021)	RCT	N = 20 men Age: 42.7 ± 1.5 Control: 9 Intervention: 9 Losses: 2	Subjects participated for 4 weeks in a familiarization program before starting the intervention. Participants were organized into 2 groups: Control group: a non-ketogenic diet was followed. Intervention group: a ketogenic diet was followed. Both groups carried out 4 strength training sessions per week for8 weeks. Upper and lower limbs strength were trained every week.	Bloodsamples Body composition Maximal strength	Both groups lost a similar amount of lean body mass and fat mass (*p* = 0.001) but preserved their maximal upper and lower limbs strength (*p* > 0.05). Basal and free testosterone increased in both groups (*p* < 0.01). Insulin levels decreased significantly in both groups (*p* < 0.01).
Zajac et al. (2014)	Cross design Uncontrolled study	N = 8 men Age: 28.3 ± 3.9 Intervention A: 4 Intervention B: 4 Losses: 0	Both groups performed moderate and intense cycling exercises for 3 days preceded by 4 weeks of carrying out the assigned diet. Intervention A had a mixed diet. Intervention B had a low-carb ketogenic diet. After a month with the assigned diet and a week of rest, the groups interchange their diets.	Biochemical analysis. VO2 peak. Body composition.	High volume training in a ketogenic diet increases fat metabolism during exercise, observable by low triglyceride levels during max effort training and resting (*p* = 0.001). The ketogenic diet reduces body mass (*p* = 0.011), fat content (*p* = 0.001) and post-exercise muscle damage, which was observed by lower rest and exercise plasma creatine kinase and lactate dehydrogenase. The ketogenic diet reduces physical performance at high intensities evidenced by a lower lactate concentration (*p* = 0.001).
Zinn et al. (2017)	Pilot study Uncontrolled or randomized study	N = 5 (4 women and 1 man) Intervention: 5 Age: 49 to 55 Losses: 0	The group continued with their usual training and also performed endurance events for 10 weeks. The intervention consumed <50 g of carbohydrates per day, 1.5 g of protein per kilo and freedom to consume the desired amount of fat.	Sports performance test. Body composition.	Decreased sports performance, visible by a decrease in time to exhaustion (*p* = 0.004). Reduction in body fat, measured by skin folds (*p* = 0.001). Improvements in the well-being of the subjects (any *p*-value was calculated).

## Data Availability

Data availability can be asked for the corresponding author.

## Abbreviations

TRF	Time-restricted feeding
QUALSYT	Standard Quality Assessment Criteria for Evaluating Primary Research Papers from a Varietyof fields
IVS	PEDro Validity Internal Scale
PEDro	Physiotherapy Evidence Database
